# Attenuation of *Pseudomonas aeruginosa* biofilm formation by Vitexin: A combinatorial study with azithromycin and gentamicin

**DOI:** 10.1038/srep23347

**Published:** 2016-03-22

**Authors:** Manash C. Das, Padmani Sandhu, Priya Gupta, Prasenjit Rudrapaul, Utpal C. De, Prosun Tribedi, Yusuf Akhter, Surajit Bhattacharjee

**Affiliations:** 1 Department of Molecular Biology & Bioinformatics, Tripura University (A Central University), Suryamaninagar, Tripura, 799022, India; 2 Centre for Computational Biology and Bioinformatics, School of Life Sciences, Central University of Himachal Pradesh, Shahpur, Himachal Pradesh, 176206, India; 3 Department of Chemistry, Tripura University (A Central University), Suryamaninagar, Tripura, 799022, India; 4 Department of Microbiology, Assam Don Bosco University, Guwahati, Assam 781017, India

## Abstract

Microbial biofilm are communities of surface-adhered cells enclosed in a matrix of extracellular polymeric substances. Extensive use of antibiotics to treat biofilm associated infections has led to the emergence of multiple drug resistant strains. *Pseudomonas aeruginosa* is recognised as a model biofilm forming pathogenic bacterium. Vitexin, a polyphenolic group of phytochemical with antimicrobial property, has been studied for its antibiofilm potential against *Pseudomonas aeruginosa* in combination with azithromycin and gentamicin. Vitexin shows minimum inhibitory concentration (MIC) at 260 μg/ml. It’s antibiofilm activity was evaluated by safranin staining, protein extraction, microscopy methods, quantification of EPS and *in vivo* models using several sub-MIC doses. Various quorum sensing (QS) mediated phenomenon such as swarming motility, azocasein degrading protease activity, pyoverdin and pyocyanin production, LasA and LasB activity of the bacteria were also evaluated. Results showed marked attenuation in biofilm formation and QS mediated phenotype of *Pseudomonas aeruginosa* in presence of 110 μg/ml vitexin in combination with azithromycin and gentamicin separately. Molecular docking of vitexin with QS associated LuxR, LasA, LasI and motility related proteins showed high and reasonable binding affinity respectively. The study explores the antibiofilm potential of vitexin against *P. aeruginosa* which can be used as a new antibiofilm agent against microbial biofilm associated pathogenesis.

Biofilms are microbial communities of surface-attached cells enclosed in extracellular polymeric substances (EPS), which is self produced. Most microbial cells live in natural environment in biofilm form due to their better adaptiveness in a given habitat^1^,^2^. For homogenous biofilm development, the microorganism efficiently attaches itself to a substratum for growth and development processes^3^,^4^. Biofilm formation from planktonic microorganism often enhances the pathogenic capability of organism. Biofilms have been found to be involved in 80% of all microbial infections in the body^5^. Biofilm associated microbial infections include urinary tract infections, catheter infections, formation of dental plaque, gingivitis, and cystic fibrosis^2^. Among biofilm forming microbial pathogens, *Pseudomonas aeruginosa* is considered to be the most sinister pathogen causing biofilm on human host. *Pseudomonas aeruginosa* causes urinary tract infections, kidney infections, cystic fibrosis (the most common life-threatening hereditary disease) etc^2^. Organisms in biofilm forms, exhibit different physiology and phenotype in comparison to their planktonic forms^3^,^4^. It was reported that the organisation of biofilm into such complex structures is regulated by cell to cell communication known as quorum sensing^6^,^7^. Several literatures documented that microbial biofilm formation generated an effective defense against a variety of stresses such as antibiotics and disinfectants^4^. Keeping in view with the growing influence of drug resistance, investigations on natural compounds have attracted considerable interest as a new class of antimicrobial compounds with antibiofilm activity^4^,^8^.

Polyphenols are secondary metabolites ubiquitously distributed in all higher plants, which play an important role in defense from a variety of microbial pathogens^9^. In regard to the chemical structure, they comprise of a wide variety of molecules with polyphenol structure and are generally divided into flavonoids and non-flavonoids^9^,^10^. Flavonoids share a common carbon skeleton of diphenyl propanes, two benzene rings (ring A and B) joined by a linear three-carbon chain. The central three-carbon chain forms a closed pyran ring (ring C) with a benzene ring. More than 4000 flavonoids have been identified in fruits, vegetables, and plant-derived beverages, such as tea and wine, and the list is constantly growing^11^. Depending on the oxidation state of the central pyran ring, flavonoids can themselves be subdivided into many subclasses such as flavonols, flavones, anthocyanidins and isoflavones^10^,^12^. For the present study, we have selected one flavone compound vitexin, isolated from plants of Vitex species due to its existing application as antimicrobial agent against variety of microorganisms^13^. We have tried to assess the effect of vitexin on modulation of microbial biofilm formation. We have also evaluated the effect of a combination of azithromycin and gentamicin separately with vitexin. It was observed that vitexin showed significant antimicrobial and antibiofilm activities against *P. aeruginosa* which was further validated by using *in vivo* catheter associated murine biofilm model. The present report explores the potentials of a flavone compound vitexin as an efficient agent to fight against *P. aeruginosa* biofilm. This study further explores the synergistic effect of azithromycin and gentamicin separately with vitexin against *P. aeruginosa*.

## Results

### Antimicrobial activity of vitexin, azithromycin and gentamicin

Since flavone compounds were reported to possess antimicrobial activity against several microorganisms^13^, so attempts were made to assess the antimicrobial activity of vitexin against *P. aeruginosa*. Antimicrobial activity of vitexin, azithromycin and gentamicin against *P. aeruginosa* were evaluated through detection of MIC by broth micro-dilution assay^14^. The MIC value of vitexin against *P. aeruginosa* was 260 μg/ml whereas azithromycin and gentamicin shows MIC at 55 μg/ml and 10 μg/ml respectively (Supplementary Table 1). This indicates that vitexin has moderate antimicrobial activity against *P. aeruginosa*. We have also determined the minimum biofilm eradication concentration (MBEC) of vitexin, azithromycin and gentamicin against *P. aeruginosa* biofilm. The determined MBEC of vitexin, azithromycin and gentamicin were >3570 μg/ml, >2950 μg/ml and >1490 μg/ml, respectively (Supplementary Table 1).

### Synergistic antibiofilm activity of vitexin in combination with antibiotics

**Figure 1:**
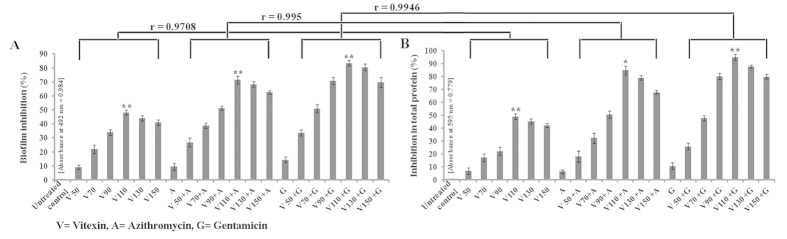
Effect of sub-MIC doses of vitexin (V), azithromycin (A) and gentamicin (G) alone and in combination of vitexin-azithromycin and vitexin-gentamicin upon percentage of biofilm inhibition (with respect to untreated control) by *P. aeruginosa* (**A**) and quantity of percentage biofilm total protein inhibition (with respect to untreated control) of *P. aeruginosa* (**B**). Each value is the average of triplicate assay where presented data is mean ± SD. Statistical analysis were done using one way ANOVA and * express P value < 0.01 and ** express P value < 0.001 (n = 3). Correlation study between experimental sets was done through Pearson’s correlation and expressed by r value.

Since vitexin exhibits antimicrobial property against *P. aeruginosa*, we hypothesize that vitexin may show antibiofilm activity against *P. aeruginosa* as well. Antibiofilm activity of vitexin against *P. aeruginosa* was evaluated at several sub-MIC doses [150 μg/ml, 130 μg/ml (½ of MIC), 110 μg/ml, 90 μg/ml, 70 μg/ml, 50 μg/ml] and in combination with sub-MIC dose (1/4^th^ of MIC) of azithromycin (13.75 μg/ml) and gentamicin (2.5 μg/ml) separately (Supplementary Table 1). Safranin staining results showed that vitexin exerted moderate biofilm attenuation against the microorganism where maximum (56%) reduction in biofilm formation was found at 110 μg/ml dose (Fig. 1A). Antibiofilm activity of vitexin increased significantly in combination with either azithromycin or gentamicin where maximum synergistic reduction (83.44%) was observed in combination of gentamicin and vitexin (Fig. 1A). Selected sub-MIC doses of vitexin, azithromycin and gentamicin were separately tested on the planktonic cells of *P. aeruginosa* to evaluate their antibacterial activity. The result showed that vitexin, azithromycin and gentamicin did not show any antimicrobial activity against planktonic form of *P. aeruginosa* at sub-MIC dose (Supplementary Figure 1).

**Figure 2:**
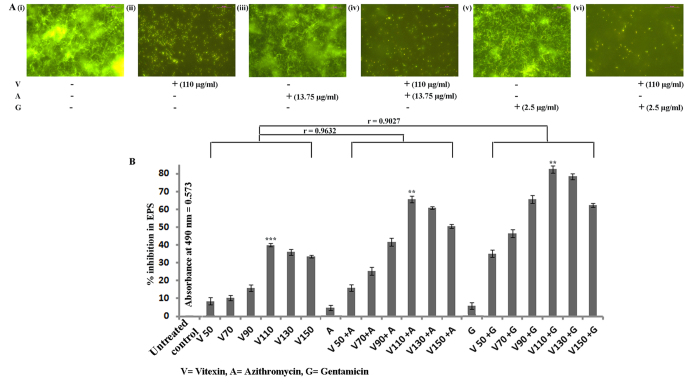
Synergistic antibiofilm effect of azithromycin and gentamicin separately on vitexin upon *P. aeruginosa*. After treatment biofilm were stained with acridine orange and observed under fluorescence microscope. Maximum effect of treatment with respect to untreated control are shown here (**A**). Each experiment was performed using three independent cultures; one representative data set is shown here. EPS quantity of *Pseudomonas aeruginosa* biofilm after treatment with vitexin alone, combination of vitexin-azithromycin and vitexin-gentamicin were estimated UV-vis spectrophotometrically and expressed as percentage inhibition (with respect to untreated control) (**B**). Each value is the average of triplicate assay where presented data is mean ± SD. Correlation study between experimental sets was done through Pearson’s correlation and expressed by r value.

Biofilm total protein assay showed that vitexin treated samples had significantly less (maximum 56.81% inhibition at 110 μg/ml dose) extracted protein in comparison to untreated control (Fig. 1B). Combination of azithromycin and gentamicin separately with vitexin led to significant attenuation in biofilm total protein (more than sum of their individual percentage inhibition). These heightened attenuation may be regarded as synergistic in nature as 110 μg/ml vitexin, with 13.75 μg/ml azithromycin showed 84.94% reduction, while with 2.5 μg/ml gentamicin showed 95.1% reduction in biofilm total protein (Fig. 1B). This observation was consistent with the safranin staining assay which was justified by >0.9 correlation coefficient between the two experimental sets (Fig. 1A,B). Further, *P. aeruginosa* was treated as per the scheme, stained with acridine orange and observed under fluorescent microscope. Images showed significant decrease in attachment of biofilm forming cells at 110 μg/ml concentration as observed from safranin staining and biofilm total protein extraction assay (Fig. 2A). In untreated control set, very prominent biofilm over glass surface can be seen (Fig. 2A). This indicates that vitexin itself can modulate *P. aeruginosa* toward development of biofilm on the glass surface. This antibiofilm activity of vitexin increased synergistically in combination with sub-MIC dose of gentamicin (maximum synergism) followed by azithromycin.

Upon extraction and quantification of EPS it was found that, in consistence with earlier observations vitexin significantly attenuated *P. aeruginosa* EPS formation where maximum 40% attenuation was seen at 110 μg/ml concentration (Fig. 2A) with respect to untreated control (OD of untreated control at 490 nm was 0.573). This degree of attenuation was synergistically and significantly increased by combination with azithromycin (65.6%) and gentamicin (82.4%) (Fig. 2A). These results indicate that vitexin at its sub-MIC level exhibited moderate biofilm attenuation activity which further increased synergistically in combination with either azithromycin or gentamicin. This synergistic effect of azithromycin and gentamicin on vitexin has been proved by determination of FIC index (Table 1). Based on above all antibiofilm study we have selected 110 μg/ml concentrations for all further sets of study to check attenuation of virulent factors.

### Attenuation of Quorum Sensing associated factors

In order to understand the underlying mechanism of biofilm attenuation of *P. aeruginosa* by vitexin and in combination, we have examined the effect of vitexin on QS mediated swarming motility, azocasein degrading protease activity, secretion of virulent factors such as pyoverdin, pyocyanin, LasA protease and LasB elastase activity.

**Figure 3:**
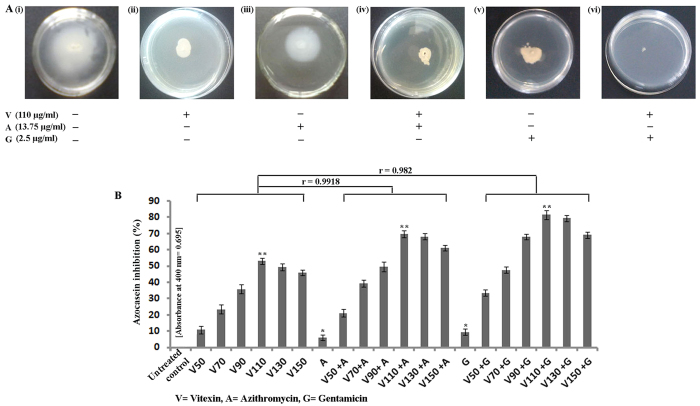
Effect of vitexin, azithromycin and gentamicin on swarming motility of *P. aeruginosa* (**A**). Each experiment was performed using three independent cultures; one representative data set is shown here. Percentage increase (with respect to untreated control) in inhibition of azocasein degrading protease release by *P. aeruginosa* is presented here (**B**). Each value is the average of triplicate assay where presented data is mean ± SD. Correlation study between experimental sets was done through Pearson’s correlation and expressed by r value.

Swarming motility leads to rapid bacterial translocation that promotes efficient colonization of the cells on a surface^4^. We have found that vitexin treated cells showed very less swarming motility in comparison to untreated control. Among all selected sub-MIC doses 110 μg/ml concentration shows maximum motility reduction (Fig. 3A). Furthermore, 110 μg/ml dose of vitexin in combination with either azithromycin or gentamicin significantly reduced the swarming motility (Fig. 3A). Bacterial proteases are hydrolytic enzymes that cleave the proteins of host cells (infected tissue), thereby facilitating bacterial invasion and growth^15^. In the present study vitexin executed moderate attenuation of bacterial protease production by *P. aeruginosa* (Fig. 3B). Furthermore, azithromycin and gentamicin synergistically reduced the motility in combination with vitexin (Fig. 3B).

**Figure 4:**
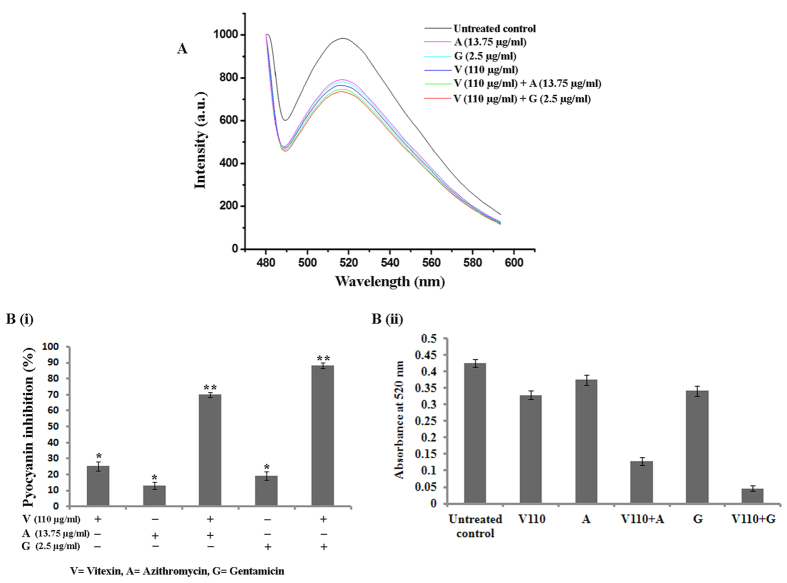
Effect of vitexin, combination of vitexin-azithromycin and vitexin-gentamicin on virulent factor production by *P. aeruginosa*. After treatment released pyoverdin were detected by fluorescence spectrophotometer and presented with respect to untreated control (**A**). Each experiment was performed using three independent cultures; one representative data set is shown here. Pyocyanin were detected by UV-vis spectrophotometer and presented as percentage inhibition (with respect to untreated control) (**B**(i)) and absorbance values at 520 nm (**B**(ii)). Each value is the average of triplicate assay where presented data is mean ± SD.

**Figure 5:**
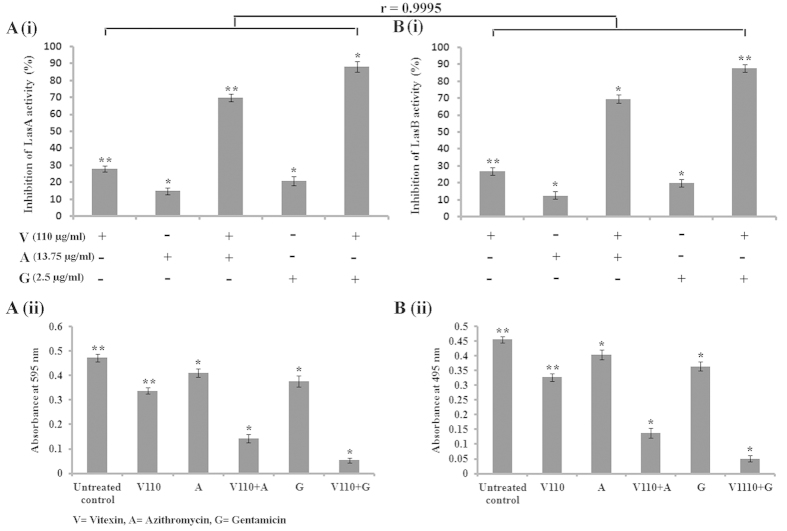
Effect of vitexin, combination of vitexin-azithromycin and vitexin-gentamicin on LasA and LasB activity of *P. aeruginosa*. After treatment LasA (**A**) and LasB (**B**) were estimated by UV-vis spectrophotometer from culture supernatant and expressed as percentage inhibition (with respect to untreated control) (**A**(i)**,B**(i)) and absorbance values (**A**(ii)**,B**(ii)).

Pyoverdin and pyocyanin are virulent factors produced by *P. aeruginosa* which are involved in bacterial virulence and pathogenesis as well. Upon treatment with vitexin, it was observed that 110 μg/ml concentration shows moderate attenuation of pyoverdin and pyocyanin. Azithromycin and gentamicin separately execute synergistic attenuation of secretion of pyoverdin (Fig. 4A) and pyocyanin (Fig. 4B) when used in combination with vitexin. In this case, gentamicin executes maximum synergistic effect. In light of the promising results obtained from total proteolytic assay, we further assessed the effect of vitexin alone and in combination in inhibiting the LasA protease activity of *P. aeruginosa*. Upon treatment with vitexin alone, we observed maximum 39.04% inhibition and in combination, a significant reduction in LasA protease activity was observed. Amongst synergistic effects, combination of vitexin and gentamicin showed better attenuation (88.33%) than combination of vitexin and azithromycin (69.93%) (Fig. 5A). Moreover, LasB elastase is also of particular interest because of its ability to produce corneal ulcers, necrotic skin lesions and pulmonary haemorrhages^16^. LasB elastase is a zinc metalloprotease capable of destroying or inactivating a wide range of biological tissues and immunological agents. Significant decrease in LasB elastase activity was observed in the culture supernatant of *P. aeruginosa* after treatment with vitexin alone (37.54%) and in combination with azithromycin (69.23%) or gentamicin (87.63%) (Fig. 5B). In consistent with LasA protease activity here also combination of 110 μg/ml vitexin and with 2.5 μg/ml gentamicin showed maximum synergistic reduction of LasB elastase activity (Fig. 5A).

### Mechanism of inhibition of quorum sensing regulator proteins of *P. aeruginosa*

**Figure 6:**
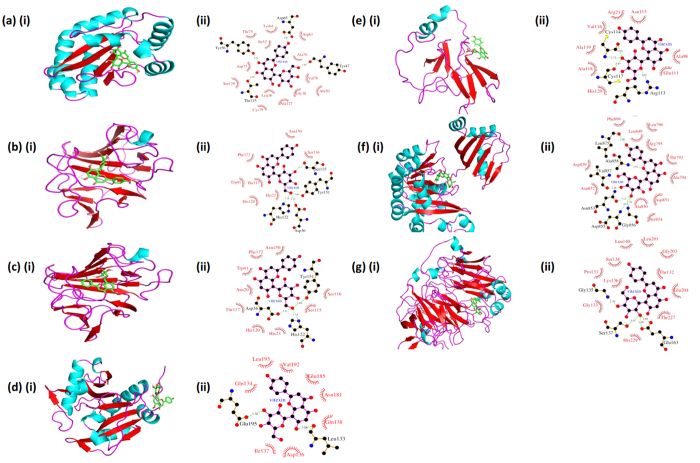
Vitexin docked in different binding pockets of QS and proteins of *P. aeruginosa*: Cartoon representation of protein-ligand complexes with helices coloured in cyan, beta strand in red and coils in magenta colour. Ligand was coloured green and represented in sticks model. Ligplots of protein-ligand complexes are showing interaction of vitexin with protein residues. (**A**) (i) Cartoon structure of LuxR with vitexin, (ii) ligplot of LuxR-vitexin complex showing interactions between protein and ligand. (**B**) (i) Cartoon structure of LasA with vitexin, (ii) ligplot of LasA-vitexin complex showing interactions between protein and ligand. (**C**) (i) Cartoon structure of LasA with vitexin, (ii) ligplot of LasA-vitexin complex showing interactions between protein and ligand. (**D**) (i) Cartoon structure of LasI with vitexin, (ii) ligplot of LasI-vitexin complex showing interactions between protein and ligand. (**E**) (i) Cartoon structure of modelled PilB protein with vitexin, (ii) ligplot of PilB-vitexin complex showing interactions between protein and ligand. (**F**) (i) Cartoon structure of PilY1 with vitexin, (ii) ligplot of PilY1-vitexin complex showing interactions between protein and ligand. (**G**) (i) Cartoon structure of PilT with vitexin, (ii) ligplot of PilT-vitexin complex showing interactions between protein and ligand.

Our studies on role of vitexin in inhibition of biofilm formation in the case of *P. aeruginosa* indicates that it is effective at sub-MIC dose and plays significant role in the attenuation of the biofilm. For further analysis of its role and to get an idea of its particular target protein, we investigated the binding affinity of vitexin to different proteins involved in the biofilm formation, QS and other phenotypes of QS such as motility of the bacteria. We have found crystal structures of three proteins related to QS, i.e. LasA (PDB ID: 3IT7), LuxR (PDB ID: 3JPU) and LasI (PDB ID: 1RO5); two motility related proteins i.e. PilY1 (PDB ID: 3HX6) and PilT (PDB ID: 3JVV). PilA and PilB did not have any crystal structure available so tertiary structure of PilB was deducted by homology modelling using a zinc binding methionine sulfoxide reductase structure (PDB ID: 2K8D) from *Methanothermobacter thermoautotrophicus* as a template. The yielded protein model was used for the docking analysis. As LasI did not have having any ligand in its crystal structure, so binding positions of the ligand from its homologous protein acyl-homoserinelactone synthase (EsaI) (PDB ID: 1K4J) from *Pantoea stewartii* subsp. *stewartii*, which was available in complex with perrhenate ions (O_4_Re) was used. For PilB the binding positions of zinc ion from homologous template protein msrB (PDB ID: 2K8D) was used for the docking studies. After docking vitexin in to the original ligand binding pockets of these proteins, we observed that vitexin is binding at the similar position as that of their native ligand in the case of protein LuxR (Fig. 6A) and LasA (Fig. 6C) with high binding scores. In the case of LasI it occupies a binding position different from the original binding pocket (Fig. 6D). Vitexin was docked with LasA at two different binding pockets occupied by two different ligands i.e. Tartaric acid (Fig. 6C) and Glycerol (Fig. 6B) respectively in the available PDB structure (PDB ID: 3IT7). In the case of motility related proteins encoded by *pil* operon, the vitexin was docked into the native binding pocket of PilY1 (Fig. 6F) and PilT (Fig. 6G), While for PilB (Fig. 6E) it was docked in to similar binding pocket as that of zinc ion in template protein msrB from *M. thermoautotrophicus* but the binding affinity was very low. Further the energy was minimized for all vitexin-protein complexes to confirm the stability of complexes and it was also observed that all complexes were having a significantly low potential energies. After energy minimization it was observed that LasI-vitexin complex in perrhenate binding pocket showed lowest potential energy as compared to the other complexes (Table 2).

### Evaluation of *ex vivo* cytotoxicity of vitexin alone and in combination with antibiotics

**Figure 7:**
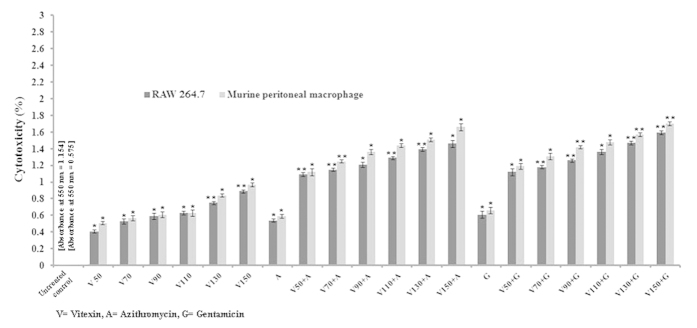
Cytotoxic effect of vitexin alone and in combination against RAW 264.7 murine macrophage cell line and murine peritoneal macrophage. Data are expressed as percentage cytotoxicity with respect to untreated control. Each value is the average of triplicate assay where presented data is mean ± SD. Statistical analysis were done using one way ANOVA and *express P value < 0.01 and **express P value < 0.001 (n = 3).

For any therapeutic drug candidate it is very important to assay its cytotoxicity on the host. To address this issue, we have treated the sub MIC doses of vitexin alone and in combination with sub MIC doses of azithromycin and gentamicin separately. The obtained results indicated that, the highest sub MIC dose of vitexin showed 0.89% of cytotoxicity in RAW 264.7 macrophages and 0.97% of cytotoxicity in murine peritoneal macrophages which is well below the permissible limit. Similarly, combination of the highest sub MIC dose vitexin with azithromycin and gentamicin separately showed cytotoxicity of 1.46% (RAW 264.7), 1.66% (murine peritoneal macrophage) and 1.59% (RAW 264.7), 1.7% (murine peritoneal macrophage) respectively (Fig. 7). All of these results indicated that the compound vitexin alone and in combination exerts negligible cytotoxicity on the host macrophages.

### Therapeutic efficacy of vitexin alone and in combination for treating catheter-associated infection in a murine model

**Figure 8:**
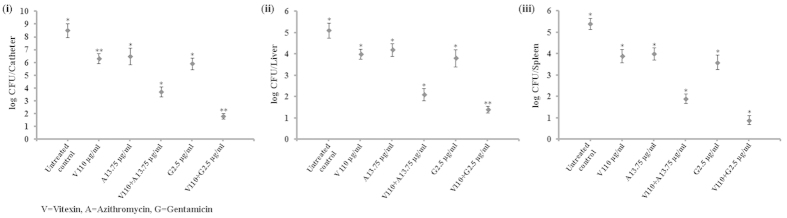
Effect of vitexin alone and in combination with azithromicin, gentamicin against *in vivo* catheter associated *P. aeruginosa* biofilm model. Data are expressed as log_10_CFU for isolated catheter (i), liver (ii) and spleen (iii). Each value is the average of triplicate assay where presented data is mean ± SD. Statistical analysis were done using one way ANOVA and *express P value < 0.01 and **express P value < 0.001 (n = 3).

A mouse model of catheter infection was used to evaluate the *in vivo* antibiofilm activity of vitexin alone and in combination with antibiotics. Bacteria were cultivated *in vitro* on implantable catheters and induced to form biofilm in mice. The effects of vitexin alone and in combination on the catheter biofilms *in vivo* are shown in Figure 8(A–C). Vitexin (110 μg/20 gm body weight) treatment plate shows log_10_6.3 cfu/ml in comparison with untreated control where cell count were log_10_8.5 cfu/ml in the catheter. This validates the antibiofilm activity of vitexin against catheter associated *in vivo* biofilm form of infection by *P. aeruginosa*. This was also observed that combination of vitexin (110 μg/20 gm body weight) with gentamicin (2.5 μg/20 gm body weight i.e. 125 μg/Kg body weight) treatment shows highest activity (log_10_1.8 cfu/ml in the catheter) in removing surface adhered bacteria. Whereas, treatment with vitexin (110 μg/20 gm body weight) and azithromycin (13.75 μg/20 gm body weight i.e. 687.5 μg/Kg body weight) also reduced bacterial load (log_10_3.7 cfu/ml in the catheter) significantly with respect to treatment with vitexin alone. Similar pattern of antibiofilm activity was also observed in case of isolated liver and spleen. All these observation firmly validates the antibiofilm activity of vitexin against *P. aeruginosa* biofilm. Results also confirm that azithromycin and gentamicin synergistically potentiates the activity of vitexin where maximum synergism was observed in combination of vitexin with gentamicin.

## Discussion

Biofilm is a densely packed community of microbial cells that attach and grow on living or nonliving surfaces and surround themselves with secreted polymers. Biofilm associated infections are often difficult to treat because of multi drug resistance, so it is important to identify new and effective molecules against bacterial biofilm formation^4^. Also, inhibition of biofilm is the first line of defence mechanism which controls surface adhered bacterial population growth and survival. Since vitexin was reported to have antimicrobial activity^17^, in the current study, we have examined the antibiofilm activity of vitexin against model biofilm forming pathogenic organism *P. aeruginosa*. We have also evaluated the effect of sub-MIC doses of conventional antibiotics such as azithromycin and gentamicin on antibiofilm activity in combination with vitexin against *P. aeruginosa*.

Vitexin is a polyphenolic flavone class of secondary metabolite ubiquitously distributed in all higher plants. In plants, polyphenolic secondary metabolites execute a defensive role against a variety of microbial pathogens^9^. Keeping this in mind, we have measured the MIC value of vitexin and also azithromycin, gentamicin against *P. aeruginosa*. As MIC dose completely kill the bacteria, there will be no chance of forming biofilm and the subsequent study of antibiofilm activity will be invalid. So, sub-MIC doses of vitexin (150 μg/ml, 130 μg/ml, 110 μg/ml, 90 μg/ml, 70 μg/ml and 50 μg/ml) were selected for examining their effect on modulation of biofilm formation by the organism (Supplementary Table 1). The antibiofilm potential of vitexin (sub-MIC doses) alone and in combination with sub-MIC dose (1/4 of MIC) of antibiotics (i.e. azithromycin and gentamicin) was estimated by performing a series of experiments. To identify the minimum concentration which can eradicate the preformed biofilm, MBEC for vitexin, azithromycin and gentamicin was determined against *P. aeruginosa* preformed biofilm^1^ (Supplementary Table 1). *P. aeruginosa* is a Gram-negative organism, so, according to Gram staining principle we have used safranin for biofilm staining. The results from safranin staining assay showed that the sub-MIC doses of vitexin had moderate antibiofilm activity against the microorganism. Combination of vitexin (sub-MIC doses) with either azithromycin or gentamicin showed synergistic antibiofilm activity where maximum attenuation was observed with the combination of vitexin (110 μg/ml) and gentamicin (2.5 μg/ml) (Fig. 1A). The biofilm total protein is a significant way to indirectly estimate the microbial biomass within the biofilm. We observed that the total protein from the experimental sets, where the organism was treated with vitexin (alone and in combination), was significantly reduced when compared with untreated control (Fig. 1B). The observation of adhered biofilm on glass surface under fluorescence microscope and quatification of EPS is also a significant method to estimate the presence of formed biofilm^18^,^19^. The results of these experiments (Fig. 2A,B) also reported a similar trend with the previous experiments. All these observations suggest that vitexin at its sub-MIC doses exerts moderate attenuation in biofilm forming capability of *P. aeruginosa*. Synergistic activity of azithromycin and gentamicin separately with vitexin has also been characterized by determination of FIC index (Table 1). The combination of vitexin and gentamicin shows a very significant synergistic FIC index of 0.078 while the vitexin-azithromycin combination shows significant synergistic FIC index 0.183^20^. Combinations of vitexin with both azithromycin and gentamicin were selected to address the issue of multi drug resistance (MDR). This has been reported in many literatures that repeated exposure of any compound or drug against a microorganism may lead to development of MDR^21^,^22^. Azithromycin and gentamicin are known antibiotic against *P. aeruginosa* and has been in use for several years^23^,^24^. Consequence of the fact is that, because of repeated exposure, *P. aeruginosa* may develop MDR against these antibiotics. In this present study, it was found that vitexin potentiates the antibiofilm activity of azithromycin and gentamicin. From this observation this can be inferred that combination of vitexin with azithromycin or gentamicin will not allow the organism to develop MDR as vitexin will potentiate the antibiofilm activity of these antibiotics against *P. aeruginosa*.

Having addressed the antibiofilm activity of vitexin (alone and in combination), we further tried to explore the underlying mechanism of biofilm attenuation. It has been reported that bacteria use a cascade of signalling events such as quorum sensing (QS) to modulate certain behaviours such as biofilm formation and virulence potentiality^17^. Attachment is the initial event in microbial biofilm formation which is positively influenced by swarming motility^4^. Swarming motility is a particular form of flagella-driven surface motility displayed by several bacteria including *P. aeruginosa*^4^. There are various plant derived compounds which have been reported to interfere with the QS signalling of microorganisms^25^. We have investigated the effect of vitexin (alone and in combination) on QS mediated phenotypes such as swarming motility (Fig. 3A), azocaseinolytic activity (Fig. 3B), release of virulent factors such as pyoverdin (Fig. 4A) and pyocyanin (Fig. 4B), LasA protease activity (Fig. 5A) and LasB elastase activity (Fig. 5B). Virulence factors are known to play an important role during the invasion of bacterial cells into the host^7^. It was reported that *P. aeruginosa* secretes proteases as virulent factors for the progression of pathogenesis^7^. Observed results of azocasein degrading assay showed that vitexin at 110 μg/ml (sub-MIC dose) moderately reduce *P. aeruginosa* protease production. Also, from all of the above experiments it can be inferred that maximum synergism was observed from the combination of sub-MIC dose (1/4^th^ of MIC) of gentamicin with vitexin.

The two QS systems, the las and rhl systems, have been found in *P. aeruginosa*^25^. The *lasI* encoded synthase that directs the formation of the diffusible signal 3O-C12-HSL molecules, which interact with the LasR transcriptional activator to activate a number of virulence genes, including *lasB*, *lasA* and *lasI* that lead to the production of various virulence enzymes such as LasB elastase, LasA protease and alkaline protease^6^,^26^. The second *P. aeruginosa* QS system (rhl) consists of rhlI, which catalyses the synthesis of the diffusible signal C4- HSL and, in conjunction with RhlR, activates expression of pyocyanin production^27^. As mentioned previously, the LasIR-encode is the most widely studied protease and elastase virulence factors which play a crucial role in pathogenesis of *P. aeruginosa*^28^. Pyoverdin is a virulence factor which competes with mammalian transferrin for iron, the successful sequestration of which essentially starves the host tissues^7^,^29^. It also promotes pathogenicity by stimulating bacterial growth^27^. Recent studies have proved that the production of siderophores such as pyoverdin is controlled through QS in *P. aeruginosa*. In our present study we have examined different sub-MIC doses of vitexin for attenuation of all these QS associated proteins and factors. We have observed that among sub-MIC dose (110 μg/ml) of vitexin exhibited moderate attenuation of QS associated proteins and factors. Furthermore combination of sub-MIC doses of either azithromycin or gentamicin separately with vitexin synergistically increased the activity. Amongst the synergistic effects, combination of 110 μg/ml dose of vitexin with 2.5 μg/ml gentamicin showed significantly maximum attenuation of QS associated proteins and factors than combination of 110 μg/ml dose of vitexin with 13.75 μg/ml azithromycin.

Further to validate QS mediated biofilm attenuation of vitexin, we have studied molecular interaction of QS associated LasA, LasI and LuxR proteins with vitexin. Our further studies on *in silico* docking analysis of vitexin with proteins involved in QS and motility showed that vitexin binds to ligand binding pockets of two of the QS proteins LasA and LuxR with a very high binding affinity, whereas in the case of LasI protein, it occupies a different position (Fig. 6). Docking studies of vitexin with motility related proteins (*pil* operon) showed that vitexin binds to PilB, PilY1 and PilT protein but the binding affinity was having very high positive values which indicated weak binding (Table 2), (Fig. 6). This indicates that the proteins related to QS may be a probable target for antibiofilm activity of vitexin. Also, to address the toxicity of vitexin (alone and in combination) against the host cells, we have performed the cytotoxicity assay in mammalian macrophage cell lines. For this study, we have used RAW 264.7 murine macrophage cell line and primary macrophages isolated from the mouse peritoneal cavity in order to correlate the toxicity between homogeneous and heterogenous phagocytic cells of the mammalian system. The results so obtained revealed that vitexin alone and in combination with azithromycin and gentamicin showed very less cytotoxicity (<2% cell lysis) against the tested macrophages (Fig. 7).

The antibiofilm effect of vitexin (alone and in combination) was further validated in an *in vivo* animal model^30^,^31^. This is known from literature that bacteria after dissociation from biofilm relocate in other organs such as secondary lymphoid organ like spleen and metabolically important organ such as liver^30^,^31^. Therefore, the bacterial load in liver and spleen is directly proportional to the dispersion from the site of catheter associated biofilm^30^,^31^. Interestingly, we have observed that treatment with vitexin (alone and in combination) reduced the bacterial load in catheter as well as in liver and spleen which are far away from implanted catheter. This suggests that treatment with vitexin executed significant inhibition on dispersion, adherence or both of bacteria to the host tissue. The *in vivo* antibiofilm activity of vitexin was synergistically potentiated by combination with azithromycin and gentamicin (Fig. 8). Therefore, from this study it can be concluded that vitexin can significantly reduce the formation of *P. aeruginosa* biofilm.

From mechanistic evaluation it can be inferred that the antibiofilm activity of vitexin is mediated through attenuation of QS process. This was also observed that anti biofilm property of vitexin can be significantly and synergistically increased by combination with sub-MIC dose (1/4^th^ of MIC) of either azithromycin or gentamicin where combination with gentamicin shows maximum activity. Thus vitexin may be projected as a potential antibiofilm agent against *P. aeruginosa,* which can further potentiate the antibiofilm activity of azithromycin and gentamicin.

## Materials and Methods

### Chemicals

Vitexin, C-glycosylated flavone was isolated and characterised from the leaves of *Vitex peduncularis* Wall (Verbenaceae) as previously mentioned by Rudrapaul *et al.*^10^.

### Microbial strain and Growth Media

*P. aeruginosa* (MTCC 2488) was used in the current study and bacteria were cultured in tryptic soy broth (TSB) media (Himedia). Initially bacteria were streaked from −80 °C glycerol stock onto tryptic soy agar (TSA) plate and single colony was inoculated into TSB media and incubated at 37 °C for 24 hrs. From there, 10^6^ CFU/mL bacterial cell suspensions were taken for all subsequent experiments.

### Animal for *in vivo* experiment

BALB/c mice (4–6 weeks old, weighting 20 g) were procured from the National Centre for Laboratory Animal Sciences, Hyderabad, India. Animals were housed in an environmentally controlled room with 12-h light–dark cycle and were kept on a standard ad libitum diet. The mice were used for experiments. All such animal experiments and protocols were carried out in accordance with the guidelines approved by the Institutional Animal Ethics Committee of Tripura University Suryamaninagar, Tripura, India. The Institutional Animal Ethics Committee of Tripura University is registered (Registration Number: 1667/GO/a/12/CPCSEA dated 12/11/2012) under The Committee for the Purpose of Control and Supervision of Experiments on Animals (CPCSEA), India.

### Determination of minimum inhibitory concentration (MIC)

MIC of vitexin, azithromycin and gentamicin against *P. aeruginosa* were determined by using standard broth micro dilution assay as outlined by CLSI^32^. Experiments were performed in triplicate.

### Evaluation of growth curve

The growth curve of *P. aeruginosa*, cultivated in the presence and absence of sub-MIC doses of vitexin, sub-MIC dose of azithromycin and sub-MIC dose of gentamicin with respect to untreated control was used for studying antibacterial activity. Briefly, standardized test inoculums (10 μl of a 10^6^ CFU/ml suspension) were added to the wells of 96 well microtitre plate already containing sub-MIC dose of compounds in 250 μl of TSB. The plates were incubated at 37 °C and the OD was recorded at 590 nm at 12 h intervals upto 48 h^33^.

### Evaluation of biofilm forming capability of *P. aeruginosa* by staining with Safranin

Before starting antibiofilm assay, the organism *P. aeruginosa* MTCC 2488 was tested for its capability to form biofilm. Briefly, *P. aeruginosa* was grown in sterile test tubes containing TSB at 37 °C for 48 h. After the incubation time, the cultures were removed and the formed biofilm was washed three times with sterile phosphate buffer saline (PBS) and stained with 0.1% (v/v) Safranin for 10 min. The excess stain was removed by washing with sterile PBS and dried overnight at 37 °C. Safranin from stained adherent *P. aeruginosa* were re-dissolved in 30% (v/v) glacial acetic acid and measured absorbance at 492 nm by UV-Vis spectrophotometer (Parkin-Elmer)^3^. The experiment was performed in triplicate.

### Determination of minimum biofilm eradication concentration (MBEC)

Biofilm was formed as described above and were then transferred to a standard 96-well plate for MBEC assay as described previously by Wu *et al.* 2014 with minor modification^30^. Briefly, biofilm was incubated overnight at 37 °C with sub-MIC doses of vitexin alone and in combination with other antibiotics. Wells were rinsed with PBS, and placed in a second 96-well plate containing vitexin alone and in combination with other antibiotics. Plate was incubated once again at 37 °C for 24 h. Viability of biofilm was then determined by measuring the turbidity at 650 nm in a 96-well plate reader (Synergy H1 Hybrid). The minimal biofilm eradication concentration (MBEC) was defined as the minimal concentration of compound required to eradicate the biofilm.

### Antibiofilm formation activity measurements of vitexin alone and in combination with either azithromycin or gentamicin

Interference of biofilm formation upon treatment with vitexin alone and in combination with either azithromycin or gentamicin was performed as the method described above. Sub-MIC doses of vitexin (150 μg/ml, 130 μg/ml, 110 μg/ml, 90 μg/ml, 70 μg/ml and 50 μg/ml) alone and in combination with sub-MIC dose of azithromycin (13.75 μg/ml) or gentamicin (2.5 μg/ml) were directly added to bacterial suspension and incubated at 37 °C for 48 h. All test tubes (treated and untreated) were then washed, stained with safranin as per the biofilm forming assay and absorbance (OD) was measured at 492 nm^3^. To find out percentage biofilm inhibition in all treated tubes with respect to untreated control, the following formula was used:


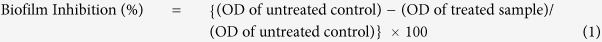


### Estimation of biofilm total protein concentration

Concentration of extractable protein was determined as the measure of *P. aeruginosa* biofilm population density on a test tube surface (treated and untreated). The microbial population density in biofilm is directly proportional to the amount of extractable protein concentration^18^. Therefore, to estimate the total extractable protein from tube surface, *P. aeruginosa* was inoculated at 37 °C for 48 h with or without sub-MIC doses of vitexin alone and in combination. After incubation, planktonic cells were removed, adhered cells in biofilm were washed gently with sterile PBS and boiled for 30 min in 5 ml of 0.5 N NaOH (Himedia, India) to extract protein. After that, the suspension was centrifuged at 10,000 rpm for 5 min and the resulting clear supernatant was collected. From supernatant protein concentration was measured according to Lowry’s method^34^.

### Microbial adherence on glass surface analysis by microscopy

To examine the effect of vitexin on biofilm formation over the glass surface, *P. aeruginosa* was separately inoculated in presence and absence of selected sub-MIC doses of vitexin alone and in combination with the antibiotics stated above. Bacteria were grown into 35 × 10 mm petridish containing sterile glass cover slips and incubated at 37 °C for 48 h. After incubation, cover slips were collected from each petridish, washed gently with sterile PBS and stained with acridine orange (4 μg ml^−1^) for 15 min in dark. Cover slips were allowed to dry and live cells in biofilm form attached on glass surface were visualised under fluorescence microscope (Leica DM 4000B, Germany)^19^. Images were captured from 20 different fields.

### Extraction and measurement of exopolysaccharide (EPS)

EPS were measured as described previously by Tribedi and Sil^18^. The biofilm formed on the glass surface was extracted in sterile water and the suspension was centrifuged at 3500 g for 20 min at 4 °C. The supernatant was collected and pellet was treated with 10 mM/l EDTA, vortexed for 15 min and re-centrifuged to extract cell-bound EPS. The supernatant was collected and mixed with the previous supernatant. The pooled supernatant was then mixed with 2.2 volume of chilled absolute ethanol, incubated at −20 °C for 1 h and centrifuged at 3500 g for 20 min at 4 °C. The pellet containing EPS was dissolved in sterile water and measured by phenol– sulphuric acid method^35^.

### Synergy studies

For interaction studies, vitexin was mixed with azithromycin and gentamicin separately to test synergistic effect of antibiotic on vitexin. This assay was performed by a checker board titration method by using 96-well polypropylene microtitre plate. The fractional inhibitory concentration (FIC) index for combinations of antimicrobials was calculated according to the equation:


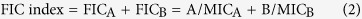


where A and B are the MICs of compound A and compound B in the combination, MICA and MICB are the MICs of compound A and compound B alone, FICA and FICB are the FICs of compound A and compound B^20^.

### Swarming motility measurement of *Pseudomonas aeruginosa*

Swarming motility of *P. aeruginosa* was investigated in small 35 × 10 mm polystyrene plates containing swarming motility media [nutrient agar (8 g/l) (Himedia, India) supplemented with glucose (5.0 g/l) (Himedia, India)]. An aliquot of 2 μl from overnight culture either treated or untreated was point inoculated in the centre of plates. Subsequently spots were allowed to dry for 20 min at room temperature and incubated at 37 °C for 48 h. Diameter of circular bacterial growth from the point of inoculation was measured^4^.

### Azocasein degrading proteolytic activity

To determine the amount of protease released by *P. aeruginosa*, the bacteria was inoculated in 24-well polystyrene plates in presence and absence of sub-MIC doses of vitexin alone and in combination and incubated at 37 °C for 48 h. The proteolytic activity was determined in the cell free culture supernatant, according to the method of Kessler *et al.*^28^ with minor modifications. Briefly, pre-incubated (either treated or untreated) cells were collected, centrifuged at 10,000 rpm for 5 min and clear supernatant were collected. 150 μl of supernatant (both treated and untreated) was added to 1 ml of 0.3% azocasein in 0.05M Tris–HCl (pH 7.5) and incubated at 37 °C for 15 min. After incubation, the reaction was stopped by the addition of 0.5 ml l0% trichloroacetic acid. Thereafter, the reaction mixture was centrifuged again at 10,000 rpm for 5 min to obtain clear supernatant. Absorbance of clear supernatant was then measured at 400 nm^14^.

### Pyoverdin secretion assay

*P. aeruginosa* were incubated in presence and absence of sub-MIC doses of vitexin alone and in combination with the antibiotics at 37 °C for 48 h. Thereafter, the culture media was centrifuged at 10,000 rpm for 15 min and the cell-free supernatant was used for pyoverdin measurement. The relative concentration of pyoverdin in all treated supernatant with respect to control was measured through fluorescence spectrophotometer at an excitation wavelength of 405 nm and an emission wavelength of 465 nm^7^.

### Pyocyanin quantification assay

The pyocyanin quantification assay was performed according to the method described by Essar *et al.*^37^. *P. aeruginosa* was incubated in presence and absence of sub-MIC doses of vitexin alone and in combination at 37 °C for 48 h and the cell-free supernatant was collected. A 5-ml culture supernatant was extracted with 3 ml of chloroform and then re-extracted with 1 ml of 0.2 N HCl to produce an orange yellow to pink coloured solution and absorbance was measured at 520 nm.

### LasA staphylolytic assay

LasA protease activity was determined by measuring the ability of *P. aeruginosa* culture supernatants to lyse boiled *Staphylococcus aureus* cells^28^. Overnight culture of *S. aureus* cells (10^6^ CFU/mL) were centrifuged at 7,000 rpm for 3 min, the pellet was suspended in 0.02 M Tris-HC1 (pH 8.5), boiled for 10 min and diluted with the same buffer to an OD of 0.8 at 595 nm. Diluted *S. aureus* suspension was added to each cell-free culture supernatant of *P. aeruginosa* (in 9:1 ratio) cultivated with or without vitexin (alone and in combination). Absorbance at 595 nm was then measured after every 15 min interval for up to 60 min.

### LasB elastase assay

The elastolytic activity of the cell-free culture supernatant of *P. aeruginosa* was determined by following the method of Ohman *et al.*^16^ by using Elastin-Congo red (ECR; Sigma, St. Louis, USA) as the substrate. One hundred (100) μl of (only vitexin as well as in combination) treated and untreated *P. aeruginosa* culture supernatant was mixed with 900 μl of ECR buffer (100 mM Tris, 1 mM CaCl_2_, pH 7.5) containing 20 mg of ECR and incubated at 37 °C for 3 h. The reaction was terminated by adding 1 ml of 0.7 M sodium phosphate buffer (pH 6.0) and the tubes were placed in cold water bath. The insoluble ECR was removed by centrifugation at 10,000 rpm for 10 min and then the absorbance was measured at 495 nm.

### Binding analysis of vitexin with quorum sensing proteins

The binding analysis was performed by scrutinising for the availability of *P. aeruginosa* QS and bacterial motility associated protein structures in the Protein Data Bank (PDB). The sdf file of vitexin (CID: 5280441) was derived from PubChem (http://www.ncbi.nlm.nih.gov/pccompound). Proteins having no available structures the PDB were modelled by Modeller 9.11^38^. Co-ordinates of native ligand binding pockets were used for docking of vitexin in the apo form of the proteins. Docking was carried out using AutoDock Vina^39^ and interactions were visualised using LIGPLOT^40^. All complexes were energy minimized using GROMACS package to confirm the stability^41^.

### Cytotoxicity assay by MTT method

Cytotoxicity of vitexin alone and in combinations was performed with murine peritoneal macrophages^42^ and RAW 264.7 macrophage cell line^43^ with minor modifications. For peritoneal macrophages, BALB/c mice were induced with intra peritoneal injection of starch (0.4%), after 48 h peritoneal macrophages were collected and cell count were identified. Side by side monolayers of RAW macrophages were also take from 60% confluent culture and cell count were identified. 2 × 10^5^ cell were seeded into each well of 96-well tissue culture plates (Eppendorf) and cultured in RPMI-1640 media supplemented with 10% FCS. Peritoneal and RAW macrophages were taken in separate 96-well plate. Cells were treated with sub-MIC doses of vitexin alone and in combination (as per the scheme of the study) and incubated in presence of 5% CO_2_ at 37 °C. RAW macrophages were incubated for 48 h whereas peritoneal macrophages were incubated for 4 hrs. Thereafter, the medium was replaced with fresh RPMI (without Phenol Red) containing 1 mg/mL MTT and incubated at 37 °C for 3 h. Followed by that formazan crystals were dissolved in DMSO by 20 min incubation at room temperature and absorbance was measured using an automatic plate reader (Synergy H1 Hybrid) at test wavelength of 550 nm.

### Mouse model of biofilm contamination by catheters

Biofilm was formed by *P. aeruginosa* MTCC 2488 on 14 gauge Teflon intravenous catheters as described previously^30^,^31^. Eight-week old specific pathogen-free female BALB/c mice, weighting 20 g, were taken and kept in several groups (3 mice in each group). Animals were housed in an environmentally controlled room with 12-h light–dark cycle and were kept on a standard ad libitum diet. Mice were anesthetized using ketamine (100 mg/kg), shoulder was shaved and disinfected with 0.5% chlorhexidine in 70% alcohol. A 4-mm incision was made longitudinally and colonized catheters containing 10^6^ cfu/mL bacteria were implanted. Subsequently, the incision was closed and disinfected with 0.5% chlorhexidine in 70% alcohol. The catheterized mice received daily caudal vein injections of vitexin alone (110 μg/20 gm body weight i.e. 5500 μg/Kg body weight) and in combination with azithromycin (13.75 μg/20 gm body weight i.e. 687.5 μg/Kg body weight), gentamicin (2.5 μg/20 gm body weight i.e. 125 μg/Kg body weight) for 3 days, starting 1 day post-colonization. Animals were sacrificed on day 4 and viable cell count was estimated. Untreated catheter implanted mice was used as control. Catheter, spleen, liver were collected upon sacrifice and homogenised tissue suspension were prepared. Tissue suspension was spread over tryptic soy agar plate, incubated for 48 hrs and cfu counts were determined^30^,^31^.

### Statistical Analysis

Each experiment was performed in triplicate to gain statistical confidence. Data values of experimental results were recorded as the mean ± standard deviation. Significance was determined by using one way ANOVA t-test and mentioned as P value < 0.01 (noted with*), P value < 0.001 (noted with**) and P value < 0.0001 (noted with***). Correlation coefficient (r) was calculated through Pearson’s Correlation method to express the correlation between different set of data. Statistical analyses were performed using Graph Pad Prism 6.0 statistical software.

## Additional Information

**How to cite this article**: Das, M. C. *et al.* Attenuation of *Pseudomonas aeruginosa* biofilm formation by Vitexin: A combinatorial study with azithromycin and gentamicin. *Sci. Rep.* **6**, 23347; doi: 10.1038/srep23347 (2016).

## Supplementary Material

Supplementary Information

## Figures and Tables

**Table 1:** Results of interaction studies between vitexin+azithromycin and vitexin+gentamicin.

Vitexin + Azithromycin		Vitexin + Gentamicin	
*P. aeruginosa* strain	FIC index	*P. aeruginosa* strain	FIC index
MTCC 2488	0.183	MTCC 2488	0.078

The FIC indexes were interpreted as follows: ≤0.5 synergy; 0.5–4.0 indifferent and >4.0 antagonism.

**Table 2:** Binding affinity and potential energy values after energy minimization of QS regulator proteins from *P. aeruginosa* with a probable inhibitor molecule vitexin.

Receptor protein	PDB ID	Native ligand	Probable inhibitor	Potential energy after energy minimization	Autodock binding score*
LuxR	3JPU	TY4	Vitexin	−5.9220925e + 05	−9.9
LasA	3IT7	Tartaric acid	Vitexin	−5.8815150e + 05	−7.8
LasA	3IT7	Glycerol	Vitexin	−5.9453588e + 05	−7.1
LasI	1RO5	perrhenate	Vitexin	−6.4595388e + 0e	−6.2

^*^Autodock gives a binding score indicating the binding affinity measured in kcal/mol. Negative scores indicate high binding affinity whereas positive scores indicate weak binding.
