# Supplementary material for: Attenuation of Pseudomonas aeruginosa biofilm formation by Vitexin: A combinatorial study with azithromycin and gentamicin
Source: Sci Rep. 2016 Mar 22;6:23347. doi: 10.1038/srep23347 (PMC4802347; doi:10.1038/srep23347)
Supplement: Supplementary Information [file srep23347-s1.pdf]

Attenuation of *Pseudomonas aeruginosa* biofilm formation by Vitexin: A combinatorial study with azithromycin and gentamicin.

Manash C. Das[1], Padmani Sandhu[2], Priya Gupta[1], Prasenjit Rudrapaul[3], Utpal C. De[3], Prosun Tribedi[4], Yusuf Akhter[2], Surajit Bhattacharjee[1*]

1. Department of Molecular Biology & Bioinformatics, Tripura University (A Central University), Suryamaninagar, Tripura, 799022, India.

2. Centre for Computational Biology and Bioinformatics, School of Life Sciences, Central University of Himachal Pradesh, Shahpur, Himachal Pradesh, 176206, India.

3. Department of Chemistry, Tripura University (A Central University), Suryamaninagar, Tripura, 799022, India.

4. Department of Microbiology, Assam Don Bosco University, Guwahati, Assam 781017, India.

***Corresponding author:**

Mailing address: Department of Molecular Biology & Bioinformatics

Tripura University, Suryamaninagar, Tripura, 799022, India

Phone: +91-03812379264

Email: sbhattacharjee@gmail.com

Table 1: Identified MBEC, MIC values of vitexin, azithromycin and gentamicin. Selection of sub-MIC doses and combinations for this study.

**Compounds**	**MBEC (µg/ml)**	**MIC (µg/ml)**	**Selected sub-MIC (µg/ml)**	**Concentrations selected for this study**					
Vitexin (V)	>3570	260	150, 130, 110, 90, 70, 50	150 µg/ml	130 µg/ml	110 µg/ml	90 µg/ml	70 µg/ml	50 µg/ml
Azithromycin (A)	>2950	55	13.75	V150 µg/ml + A 13.75 µg/ml	V130 µg/ml + A 13.75 µg/ml	V110 µg/ml + A 13.75 µg/ml	V90 µg/ml + A13.75 µg/ml	V70 µg/ml + A 13.75 µg/ml	V50 µg/ml + A 13.75 µg/ml
Gentamicin (G)	>1490	10	2.5	V150 µg/ml + G 2.5 µg/ml	V130 µg/ml + G 2.5 µg/ml	V110 µg/ml + G 2.5 µg/ml	V90 µg/ml + G 2.5 µg/ml	V70 µg/ml + G 2.5 µg/ml	V50 µg/ml + G 2.5 µg/ml

Figure legend:

Supplementary Figure 1: Effect of sub MIC doses of vitexin, sub MIC dose of azithromicin and sub MIC dose of gentamicin on *P. aeruginosa* growth pattern upto 48 hours.

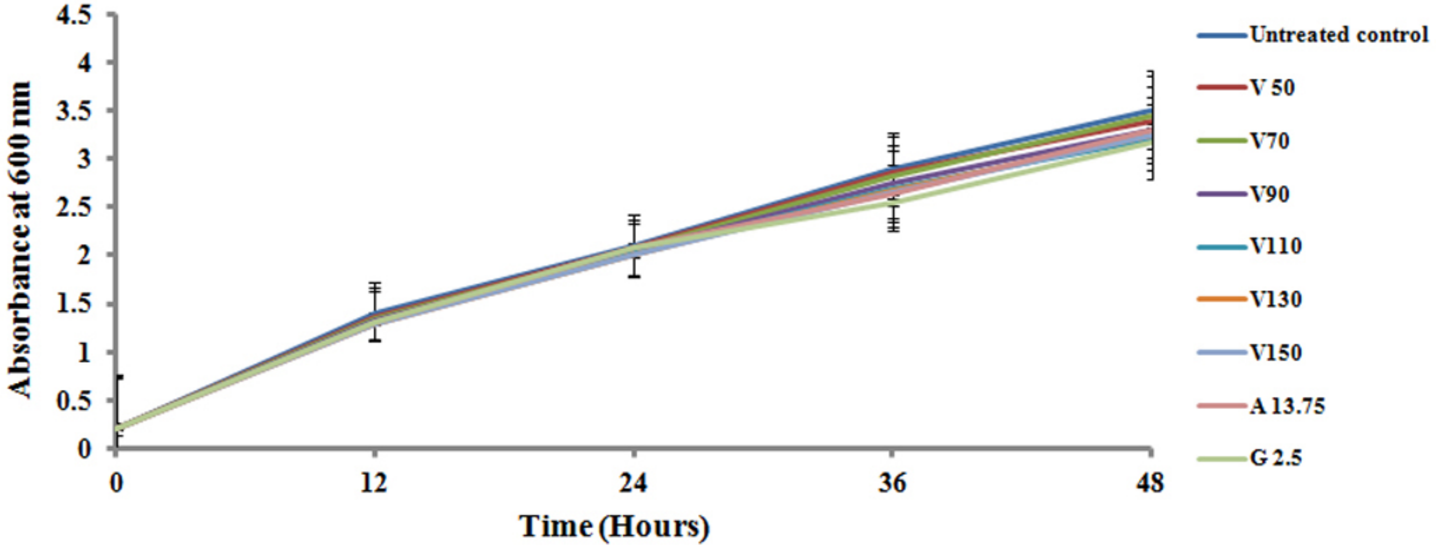

V= Vitexin, A= Azithromycin, G= Gentamicin
